# Drug-drug Interactions between Hypoglycemic and Non-hypoglycemic Medication in Diabetic Patients with Comorbidities in a Tertiary Care Center: A Descriptive Cross-sectional Study

**DOI:** 10.31729/jnma.7080

**Published:** 2021-11-30

**Authors:** Lujaw Ratna Tuladhar, Shirish Lall Shrestha, Deepak Regmi, Sneha Bimali, Srijana Bhusal, Pingala Khadka

**Affiliations:** 1 Department of Pharmacology, Nepal Medical College and Teaching Hospital, Kathmandu, Nepal; 2 Department of Internal Medicine, Nepal Medical College and Teaching Hospital, Kathmandu, Nepal; 3 Nepal Medical College and Teaching Hospital, Kathmandu, Nepal

**Keywords:** *diabetes mellitus*, *drug interactions*, *hypoglycemic agents*

## Abstract

**Introduction:**

Drug-drug interaction is one of the causes of adverse drug reactions. Generally, drug-drug interaction is common in multidrug therapy. Diabetic patients, particularly due to associated comorbidities tend to have various drug-drug interactions due to the effect of multiple drugs. The objective of this study was to find out the prevalence of drug-drug interactions in diabetic patients.

**Methods:**

It was a descriptive cross-sectional study that was conducted among previously diagnosed diabetic patients visiting the outpatient department of medicine at a tertiary care hospital between March 2021 and August 2021. Ethical approval was taken from the institutional review committee (Ref no: 030-076/077). Data was collected from diabetic patients presenting to the outpatient department of medicine using a preformed self-constructed questionnaire. Convenient sampling was done. Statistical Package for Social Sciences version 16 and Microsoft Excel were used for data analysis. Point estimate at 95% confidence interval was calculated along with frequency and proportion for binary data.

**Results:**

The prevalence of drug-drug interaction between hypoglycemic and non-hypoglycemic medication was seen in 56 (44.1%) (35.5-52.7 at 95% Confidence Interval) of the patients out of which at least one drug-drug interaction was seen in 48 (37.8%) of the patients.

**Conclusions:**

Our study showed the prevalence of drug-drug interactions in diabetic patients to be higher than other studies done in similar settings. Based on the severity, we observed two types of drug-drug interactions; close monitoring drug-drug interactions and minor drug-drug interactions.

## INTRODUCTION

Patients are commonly treated with more than one drug.^1^ This can result in drug-drug interaction (DDI) which can contribute to the failure of therapy or adverse drug reaction (ADR) which leads to an increase in the risk of hospitalization and added health care costs.^2,3^

These interactions become more substantial with multidrug therapy. Multidrug therapy is seen among diabetic patients having numerous comorbidities.

The objective of the study is to find the prevalence of drug-drug interactions between hypoglycemic and non-hypoglycemic medications in diabetic patients.

## METHODS

It was a descriptive cross-sectional study conducted among previously diagnosed diabetic patients visiting the outpatient department of medicine at Nepal Medical College and Teaching Hospital. The study was commenced from March 2021 to August 2021 for 6 months. Ethical approval was taken from the NMCTH (Ref no: 030-076/077). Written informed consent was taken from each patient. Diabetic patients with comorbidities who are exposed to at least two drugs (one hypoglycemic and other nonhypoglycemic) and wish to participate in the study were included. Diabetic patients who were admitted in the ward, patients who were on monotherapy with hypoglycemic drugs, drug-drug interactions among hypoglycemic medication, and interactions among non-hypoglycemic medications were excluded in this study. Drugs not included in the Medscape database were also excluded.

The sample size was calculated as follows,

n = Z^2^ × p × q / e^2^

  = 1.96^2^ × 0.5 × (1-0.5) / 0.09^2^

  = 119

where,

n= required sample size,
Z= 1.96 at 95% Confidence Interval (CI),
p= prevalence taken as 50% for maximum sample size,
q= (1-p)
e= margin of error, 9%

The required sample size will be 119 for the study. However, 127 patients were studied and the sampling method used was convenience sampling.

Data on patient's details and medications profile was collected from the outpatient department (OPD) of medicine by the researchers themselves by interviewing individually through the preformed self-constructed questionnaire. Pretesting was done in 10% of the sample population. The questionnaire was modified as per subject expert. Reliability of the questionnaire was maintained. Self-reported sociodemographic details, diagnosis, comorbidities, and different medications were noted by the investigator. The drug names were entered in the Medscape drug-drug interaction checker tool^5^ for various drug-drug interactions and entered in SPSS 16 for descriptive statistical analysis. Medscape contains a separate tool for detecting DDIs known as a multi-drug-drug interaction checker tool. The drugs were entered into the tool to identify potential DDI and classified them based on severity as contraindicated, serious (risk of life-threatening drug-drug interaction; use alternate), significant (potential for dangerous interaction), use with caution, monitor closely, and minor (non-significant interaction).^6^

Data entry and analysis was done using the Statistical Package for the Social Sciences version 16. Point estimate at 95% CI was calculated along with frequency and percentage for binary data.

## RESULTS

The data was collected by interviewing 127 patients who visited the outpatient department of medicine. The prevalence of drug-drug interaction between hypoglycemic and non-hypoglycemic medication was 56 (44.1%) (35.5-52.7 at 95% Confidence Interval) of the patients out of which at least one DDI was seen in 48 (37.8%) of the patients. Out of a total of 56 patients, 22 (39.28%) patients were males and 34 (60.71%) patients were females.

**Table 1:** Table showing number of drug-drug-drug interactions (n=127).

Number of drug-drug-drug interaction	n (%)
No drug-drug interaction	71 (55.9)
One drug-drug interaction	48 (37.8)
Two drug-drug interactions	6 (4.7)
Three drug-drug interactions	1 (0.8)
Four drug-drug interactions	1 (0.8)

The Medscape tool for checking drug-drug interaction classified the drug-drug interactions observed in our study into two categories: monitored closely and minor (Table 2).

**Table 2:** Table showing drug-drug interaction between hypoglycemic and non-hypoglycemic medication (n = 56).

Drug-drug interactions	n (%)	Interaction
Metformin-Amlodipine	35 (27.6)	Monitor closely
Metformin-Thyroxine	13 (10.2)	Monitor closely
Metformin-Enalapril	3 (2.4)	Monitor closely
Glimepiride-Aspirin	3 (2.4)	Monitor closely
Insulin-Telmisartan	2 (1.6)	Monitor closely
Insulin-Losartan	2 (1.6)	Monitor closely
Repaglinide-Atorvastatin	1 (0.8)	Monitor closely
Metformin-Furosemide	1 (0.8)	Minor
Metformin-Hydrochlorothiazide	1 (0.8)	Minor
Glimepiride-Hvdrochlorothiazide	1 (0.8)	Minor
Metformin-Budesonide	1 (0.8)	Minor

The commonly used medications for the management of diabetes were metformin (85.8%), glimepiride (26.8%), and insulin (18.9%). The common nondiabetic medications used for the management of various comorbidities associated with diabetes were amlodipine (33.10%), atorvastatin (29.10%), and losartan (26.80%) (Table 3).

**Table 3:** Table showing frequency and percentage of hypoglycemic and non-hypoglycemic medication (n=127).

Hypoglycemic medication		Prescribed frequency n (%)
	Metformin	109 (85.80)
	Glimepiride	34 (26.80)
	Insulin	24 (18.90)
	Linagliptin	13 (10.20)
	Sitagliptin	7 (5.50)
	Empagliflozin	5 (3.90)
	Voglibose	4 (3.10)
	Acarbose	3 (2.40)
	Teneligliptin	2 (1.60)
Non-hypoglycemic medication	Repaglinide	1 (0.80)
	Amlodipine	42 (33.10)
	Atorvastatin	37 (29.10)
	Losartan	34 (26.80)
	Rosuvastatin	30 (23.60)
	Thyroid hormone	16 (12.60)
	Telmisartan	8 (6.30)
	Aspirin	8 (6.30)
	Pantoprazole	8 (6.30)
	Budesonide	5 (3.90)
	Metoprolol	4 (3.10)
	Tiotropium	4 (3.10)
	Enalapril	3 (2.40)
	Salbutamol	3 (2.40)
	Formoterol	3 (2.40)
	Febuxostat	3 (2.40)
	Omeprazole	2 (1.60)
	Fenofibrate	2 (1.60)
	Prazosin	2 (1.60)
	Furosemide and spironolactone	1 (0.80)
	Hydrochlorothiazide	1 (0.80)
	Rabeprazole	1 (0.80)
	Ilaprazole	1 (0.80)
	Carboprost	1 (0.80)
	Dapoxetine	1 (0.80)
	Ursodeoxycholic acid	1 (0.80)
	Clonidine	1 (0.80)
	Clonazepam	1 (0.80)
	Carbimazole	1 (0.80)

## DISCUSSION

The study aimed to identify potential drug-drug interactions (DDI) between hypoglycemic and nonhypoglycemic medications in diabetic patients with comorbidities. Drug-drug interactions (DDIs) are defined as two or more drugs interacting in such a manner that the effectiveness or toxicity of one or more drugs is altered on administration of the other.^7^ DDI is characterized as a clinical event that occurs when the effect and/or toxicity of a drug are altered by the presence of another drug. The result of DDI may be positive (enhanced effectiveness) or negative (decreased effectiveness, toxicity or idiosyncrasy), they are generally unpredictable and undesirable in pharmacotherapy.^4^ We observed drug-drug interaction between hypoglycemic and nonhypoglycemic medication in 44.1% of the patients. Among the 44.1% of the patients, most of the drug-drug interaction was single drug-drug interaction (37.8%) i.e. one hypoglycemic agent having drug-drug interaction with one non-hypoglycemic drug. Most of the drug-drug interactions that were observed in our study required close monitoring while few drug-drug interactions were minor (non-significant interaction). DDI that require close monitoring demonstrate that the specified agents may interact with each other in a clinically significant manner. The benefits of concomitant use of these two medications usually outweigh the risks. An appropriate monitoring plan should be implemented to identify potential negative effects. Dosage adjustments of one or both agents may be needed. DDI that were minor or non-significant indicate that the specified agents may interact with each other, but there is little to no evidence of clinical concern resulting from their concomitant use.^7^

Drug-drug interaction between metformin-amlodipine, metformin-thyroid hormone was common (>10%) while drug-drug interactions between metformin-enalapril, glimepiride-aspirin, insulin-telmisartan, insulin-losartan, repaglinide-atorvastatin, metformin-furosemide, metformin-hydrochlorothiazide, glimepiride-hydrochlorothiazide, and metformin-budesonide were less common (<10%). Metformin when given along with amlodipine decreases the effects of metformin by pharmacodynamic antagonism. It can lead to hypoglycemia on withdrawal of amlodipine.^5^ Metformin when given along with thyroxine decreases the effects of metformin by pharmacodynamic antagonism. It can lead to hypoglycemia on withdrawal of thyroxine.^5^ Metformin when given along with enalapril increases toxicity of metformin by unspecified mechanism. There is increased risk for hypoglycemia and lactic acidosis.^5^ Glimepiride when given with aspirin increases the effect of glimepiride by plasma protein binding competition leading to the risk of hypoglycemia.^5^ Insulin when given along with telmisartan increases the effect of insulin by pharmacodynamic synergism.^5^ Insulin when given along with losartan increases the effect of insulin by pharmacodynamic synergism.^5^ Repaglinide when given along with atorvastatin increases toxicity of atorvastatin i.e. Increased risk of myopathy.^5^ Metformin when given along with furosemide decreases the effect of furosemide while furosemide increases the effect of metformin by unspecified interaction.^5^ Metformin when given along with hydrochlorothiazide decreases the effect of metformin by pharmacodynamic antagonism and hydrochlorothiazide will increase the level of metformin by competing for renal tubular clearance.^5^ Glimepiride when given along with hydrochlorothiazide decreases the effect of glimepiride by pharmacodynamic antagonism.^5^ Metformin when given along with budesonide decreases the effects of metformin by pharmacodynamic antagonism.^5^

We also observed that metformin, glibenclamide, and insulin were among the common hypoglycemic medications that were used for the management of diabetes. They were also the common medication to have a drug-drug interaction with other medications that were prescribed for comorbid conditions associated with diabetes. We observed amlodipine (33.10%), atorvastatin (29.10%), losartan (26.80%), and rosuvastatin (23.60%) as common medication used for management of common comorbidities like hypertension (55.90%), hyperlipidemia (52.8%), and hypothyroidism (13.4%).

The drugs are often used in combination to achieve a synergistic or additive action. Moreover, multidrug therapy is used when multiple disease conditions have to be treated. A study conducted by Indu R et al concluded that diabetic patients suffer from several comorbid conditions due to which the level of multidrug therapy was high.^13^ In such conditions one cannot assume that the effect of the single drug would remain the same when it is combined with the others. Some interactions can produce toxicity or inhibit the drug's efficacy or therapeutic benefit. In another study conducted by Dobrica EC concluded that polypharmacy should be an area of serious concern as diabetic patients received more drugs than their non-diabetic counterparts and were exposed to more drug-drug interactions.^14^ Drug-drug interaction should always be considered when adverse effects are seen. A study concluded that potential drug-drug interaction should be carefully investigated.^4^ Another study also reported that in a survey conducted among 1601 subjects, 46% had at least one clinically significant drug-drug interaction and 10% of those interactions were considered as highly serious.^15^ The drug-drug interaction may be pharmacokinetic (the delivery of the drug to its site of action is hampered by other drugs) or pharmacodynamic (response of the drug target is modified by the second drug). The common drug-drug interaction observed in our study was mostly pharmacodynamic interaction. Although not analyzed in this study but interaction of food with the drug also increases the risk of a food-drug interaction.^16^ The drug-drug interactions that were observed in our study need to be monitored closely. Some of the drug-drug interactions were minor or non-significant interactions. We did not observe any life-threatening, serious, or significant drug-drug interaction in our study. A study conducted by Sankar V et al^17^ had screened 50 prescriptions out of which nine drug-drug interactions were between hypoglycemic and non-hypoglycemic medication^17^ which was approximately 18%. In another study conducted by Prado M et al interviewed 1517 individuals reported 119 drug-drug interactions between hypoglycemic and non-hypoglycemic medication^4^ which is approximately 7.8%. These records indicate that drug-drug interactions were much higher in our study.

The study conducted by Sankar V et al^17^ reported the drug-drug interactions between various hypoglycemic and non-hypoglycemic drugs. The interactions between the drugs were as follows: glibenclamidediclofenac, glibenclamide-ranitidine, glibenclamidehydrocortisone, glimepiride-budesonide, glimepiride-aspirin, metformin-budesonide, insulin-aspirin, insulin-levofloxacin, and insulin-metoprolol. The study had reported cases of infection in diabetic patients following which antibiotics were used.^17^ In our study, patients have not reported the use of antibiotics therefore we have not come across such drug-drug interaction between hypoglycemic medication and antibiotics. In another study conducted by Prado M et al reported drug-drug interaction between glibenclamide-hydrochlorothiazide, glibenclamideaspirin, insulin-aspirin, metformin-propranolol, metformin-atenolol, metformin-enalapril.^4^ In our study, drug-drug interaction between metformin and amlodipine (27.6%) was the commonest. A study that was conducted on rabbits concluded that amlodipine causes hyperglycemia in normal rabbits and its use with metformin increases the hypoglycemic effect of metformin. Therefore, it should be used cautiously in diabetic patients.^18^ Similarly, another common interaction (>10%) seen in our study was between metformin and thyroxine. Metformin enhances the effect of thyroxine which is to increases blood glucose.^19^ This can lead to decreased efficacy of metformin. Other drug-drug interactions that were observed in our study were less common and some were non-significant. A study conducted by Upadhyay DK et al, reported that metformin was the anti-diabetic drug with the greatest risk of DDI and the most common drug-drug interaction observed was between metformin and enalapril. Metformin was also found to have a drug-drug interaction with atenolol and ranitidine.^2^ Antidiabetic drug gliclazide was found to have DDI with atenolol while glibenclamide was found to have DDI with atenolol and aspirin.^2^ Contradicting to the above study, another study conducted by May M concluded that metformin has very low interaction potential but caution should be taken when other drugs that impair the renal function are used. However, incretin mimetic (sitagliptin, saxagliptin) and sodium-glucose co-transporter-2 (SGLT-2: canagliflozin, dapagliflozin) inhibitors comprise of very low interaction potential and are therefore recommended as an ideal combination partner from a clinical-pharmacological point of view. A study conducted by Sheen A concluded that inhibitors of sodium-glucose cotransporter-2 were not influenced by concomitant administration of other glucose-lowering drugs or cardiovascular agents commonly used in type 2 diabetes.^20^

We came across certain limitations in this study. The soul of this study is the Medscape drug interaction checker tool. Although Medscape is a popular application as well as a popular webpage among health professionals, some drugs were not listed in Medscape database but prescribed to the patients. Interaction between such drugs and hypoglycemic medication could not be looked into. Also, interaction between drugs that patients could not recall at the time of the consultation was missed. The advantage of Medscape is that it is a free application. Another application for monitoring drug-drug interaction is IBM Micromedex drug interaction.^8,9^ Another limitation is the selection of patients. Diabetic patients who were admitted in the ward or even diabetic patients who had come for dialysis were not enrolled in this study which is a source for potential bias. However, the inclusion of dialysis patients might not represent usual diabetic patients. Another limitation of this study was that drug-drug interaction between two hypoglycemic drugs was not included as its use is intended for additive or synergistic action.

## CONCLUSIONS

Our study showed the prevalence of drug-drug interactions in diabetic patients to be higher than other studies done in similar settings. Based on the severity, we observed two types of drug-drug interactions; close monitoring drug-drug interactions and minor drug-drug interactions. We did not observe any serious or life-threatening drug-drug interaction between hypoglycemic and non-hypoglycemic medication.
